# Restaurant marketing to kids in Canada: associations with restaurant consumption and appealing restaurant advertisement features in a nationally representative sample of Canadian young people aged 9–17 years

**DOI:** 10.1017/S1368980025101262

**Published:** 2025-10-14

**Authors:** Leia Minaker, Dana Lee Olstad, Elise Pauzé, Monique Potvin Kent

**Affiliations:** 1 School of Planning, Future Cities Institute, University of Waterloo, Waterloo, Canada; 2 Department of Community Health Sciences, University of Calgary, Calgary, Canada; 3 School of Epidemiology and Public Health, University of Ottawa, Ottawa, Canada

**Keywords:** Marketing to children, Restaurants, Canada, Quebec Consumer Protection Act, Marketing appeal

## Abstract

**Objective:**

Restaurant marketing to children may be associated with consumption. We examined whether and to what extent reported frequency of restaurant advertisements exposure was associated with consumption and money spent at all types of restaurants among children living in Canada. We also describe what children and youth report as appealing restaurant marketing techniques.

**Design:**

This study reports findings from a cross-sectional, online survey. The survey covered reported exposure to restaurant marketing, restaurant product consumption, money spent at restaurants and appealing features of restaurant advertisements. Descriptive statistics and adjusted and unadjusted linear and logistic regressions were constructed.

**Setting:**

Canadian provinces

**Participants:**

1500 children and youth aged 9–17 years.

**Results:**

A third (32 %) of participants reported restaurant advertisement exposure at least once per day. Overall, 43 % of participants consumed restaurant products more than twice per week, 61 % spent at least some money at a restaurant in the last 7 d, and of those who spent money, the mean expenditure in the last week was $20·70. Frequency of advertisement exposure was significantly associated with all outcomes. Several significant differences in outcomes emerged by region, age and race/ethnicity. Pictures were the most appealing marketing technique among both age groups; however, youth (aged 13–17 years) seemed to prioritise price and price promotions, while children (aged 9–12 years) prioritised toys, humour and winning prizes.

**Conclusions:**

A large proportion of Canadian children and youth consumed restaurant offerings more than twice a week. Reported restaurant advertising exposure was significantly positively associated with restaurant consumption frequency and money spent at restaurants.

Globally, unhealthy diets are a leading cause of morbidity and mortality^(1)^, and poor diet quality persists, even in wealthy nations^(2–5)^. Environmental determinants of dietary intake – often referred to as ‘food environments’ – have long been recognised as important in shaping population eating patterns^(6,7)^. In a widely cited Model of Community Nutrition Environments^(6)^, environmental variables purported to shape dietary patterns include community, consumer and organisational nutrition environments. The community nutrition environment comprises the types and locations of food outlets available in a neighbourhood, while the consumer nutrition environment includes everything within food stores and restaurants, including food marketing (price, promotion, placement and products). Another key component of the framework is the information environment, which focuses more broadly on media and advertising in all settings^(6)^.

Children and youth worldwide commonly consume foods and beverages in restaurants. In a 32-country study with over 100 000 participants aged 12–15 years, 53 % of participants reported eating fast food at least once per week^(8)^. A more recent 54-country study found 55 % of adolescents ate fast food at least 1 d per week, and 10 % ate fast food between 4 and 7 times per week^(9)^. Frequent consumption of restaurant food is a major health concern. In Canada for example, children’s restaurant meals, much like regular menu items^(10)^, are typically of low nutritional quality. For instance, half of meals sold in Canadian sit-down restaurant chains and 35 % of those sold in fast-food restaurant chains surpassed the daily recommended intake of Na for children aged 4–8 years^(11)^. Thus, it is not surprising that on days they patronise fast-food restaurants, children consume more added sugar, total fat, Na and sugary drinks, and less fruits and vegetables compared to days they do not patronise fast-food restaurants^(12–14)^. Finally, fast-food consumption has been linked to higher BMI among children and youth both cross-sectionally^(15)^ and prospectively^(16)^.

Food and beverage marketing for restaurants may cause children and youth to visit restaurants more often. Moreover, this marketing may negatively impact their dietary intake, BMI and health^(17–22)^. Most existing research on marketing to children and youth focuses on television advertising, digital marketing, packaging and outdoor marketing to children^(17–22)^. In Canada, advertising expenditures are highest for fast-food and sit-down restaurants, and children are most exposed to fast-food advertising via television and digital media^(23–26)^. While restaurant advertising is prevalent on television and in digital media, one of the existing gaps in marketing research is the extent to which children are exposed to all restaurant food marketing (e.g. in-store, radio, outdoor signage, digital and television) rather than exclusively focusing on digital and television marketing^(20,21,27–29)^. Another major gap in understanding is the extent to which and how children and youths’ exposure to restaurant advertising is associated with their consumption of foods and beverages from restaurants^(30)^. While prior research has explored the power^(31)^ and appeal^(32)^ of food and beverage advertising features, a final research gap is that information on the kinds of restaurant-specific advertising features that are appealing to children and youth is lacking.

This study had two main objectives. The first objective was to examine whether and to what extent reported frequency of exposure to advertisements about restaurants was associated with (i) frequency of restaurant food and beverage consumption and (ii) the amount of money children spent at restaurants in the past week, among children living in Canada, and after adjusting for sociodemographic covariates. The second objective was to describe what children and youth report as appealing restaurant marketing techniques. Of note, ‘children’ are typically defined as persons up to the age of 18 years, and there is no universally agreed-upon definition of ‘youth’^(33)^. For the purposes of this paper, ‘children’ are considered 9–13 years of age (ages at which children in Canada are typically in elementary school) and ‘youth’ are considered 14–17 years of age (ages at which children in Canada are typically in secondary school).

## Materials and methods

### Study design

This study used a cross-sectional, quantitative survey design. The survey was administered by the Survey Research Centre (SRC) at the University of Waterloo and was conducted online in English and French among children and youth living in Canada who were recruited using online panels coordinated by Leger and Asking Canadians. Eligible respondents were males and females, aged 9–17 years who resided in one of the ten Canadian provinces. As all respondents were under 18 years of age, parental/guardian consent, as well as child assent, was obtained/confirmed prior to survey participation. Quotas for sex and for two age groups: (1) children aged 9–13 years, and (2) youth aged 14–17 years were applied across four Canadian regions consisting of the ten Canadian provinces: Eastern Canada (Newfoundland and Labrador, Nova Scotia, Prince Edward Island, New Brunswick), Quebec, Ontario and Western Canada (British Columbia, Alberta, Saskatchewan and Manitoba) to ensure the data collected were nationally representative in terms of age, sex and region of residence of the Canadian population, for those living in the provinces (99·7 % of the population)^(34)^. As all the quotas were successfully filled, sampling weights were not required.

### Recruitment

The web survey was launched via an email invitation sent by the panel firms inviting members who were parents or caregivers of children or youth and who satisfied the region, age and sex eligibility specifications to complete the survey. A total of 14 303 parents were invited by email to complete the survey. The cooperation rate (how likely an eligible person was to be reached and to complete the survey) was 77·6 %. The overall response rate (the number of eligible people who completed the survey, including the number of potentially eligible respondents with whom the panel firm was unable to make contact) was 14·5 %, for a total sample of 1500 respondents. Data collection took place from 8 to 28 February 2022. The response rate is in line with the response rates the SRC typically experiences for web studies (10–15 %). The survey took 6·3 min to complete, on average. Participants were compensated via standard practices of Leger and Asking Canadians panel firms, which can include draws for prizes, such as tablets and other small electronic devices.

### Survey

After providing consent to participate, parents or caregivers indicated the region in which they and their child/youth currently lived, their child’s age, sex at birth, race/ethnicity and their annual household income before taxes (all response options described below). Upon completion of these questions, the parent was instructed to ask their child/youth to complete the remainder of the survey. Upon confirming the child/youth’s informed assent, the child/youth portion of the survey began.

The child/youth survey questions were informed by findings from a prior qualitative study (unpublished data), which collected data during semi-structured interviews with twenty-seven Canadian children and youth (ages 9–17 years) that explored ways in which children and youth engaged with food marketing in restaurants. Questions were developed by the research team and edited by senior staff at the SRC at the University of Waterloo, who have expertise and substantial experience in conducting thousands of surveys with children and youth.

### Measures

#### Outcome variables

All outcome variables were derived from the child/youth portion of the survey. For research objective 1, the main outcome of interest was the *frequency of restaurant consumption,* defined as the number of days in the last 7 d participants reported eating or drinking items purchased from a restaurant (including dining in, takeout and delivery from all types of restaurants: sit-down, fast-food, cafes, etc.). Participants were asked separately about eating and drinking at restaurants, and these two variables were summed to create a measure of overall frequency of restaurant patronage (range 0–14 times in the previous 7 d). The derived variable was not normally distributed, and thus *frequency of restaurant consumption* was dichotomised as *high-frequency consumption* (more than twice in the previous 7 d) *v*. *low-frequency consumption* (two or fewer times in the previous 7 d).

Children’s responses to the question, ‘Thinking about the last seven days, about how many dollars did you spend on foods and drinks from restaurants, fast food places, or cafés?’ were used to derive two additional outcome variables. First, *restaurant spending (yes/no)* was defined as ‘yes’ if participants reported spending any money in the last 7 d, and ‘no’ if they reported spending no money. Second, *money spent at restaurants* was defined as the number of dollars participants reported spending on foods and drinks from restaurants in the last 7 d among those who reported spending money. *Money spent at restaurants* was a continuous variable that was not normally distributed, and there were outliers (i.e. > $100 in the last 7 d), which were removed from the analyses (twenty-one outliers removed from analyses). A Box-Cox transformation was carried out in SPSS to normalise the data, and *money spent at restaurants* was then treated as a continuous outcome.

For research objective 2, the outcome of interest was *appealing marketing techniques in restaurant advertisements*, where participants were asked, ‘Imagine you were walking by a restaurant and they had a sign or poster advertising their foods or drinks. What features of the poster would draw you into the restaurant the most?’ Participants could select all pre-defined response options that applied, and response option order was randomised in the survey. Response options included: Visual Features: colours, font or lettering, Pictures of the food or drink, A description of the taste (e.g. crunchy, juicy, delicious), Nutrition information, Toys or other free item with purchase, The price of the item, Whether there is a special price or ‘deal’ on the item (e.g. buy two for $3; buy one get one for $1), You can collect points if you buy this item, A funny or interesting slogan or caption, The item is vegetarian or plant-based, The item is good for the environment, You can support a good cause (like a charity or a sports team) by buying an item and You can win prizes if you get this item (e.g. enter into a draw, play Roll up the Rim or Monopoly).

#### Independent variable

Participants were asked to self-report their overall exposure to restaurant advertisements via the following question, ‘In a typical week, how often do you see or hear advertisements for fast food, restaurants, or cafés in any kind of media (e.g. online, TV, games, radio, posters, billboards, apps, or anywhere else)?’ Response options included never, less than once a week, once a week, a few times a week, every day, more than once a day and don’t know. *Frequency of advertisement exposure* was defined as: ‘Less than once per week’, ‘Once a week to a few times per week’ and ‘At least once a day’. ‘Don’t know’ responses were counted as missing for the inferential statistics, but prevalence of all response options is reported in the descriptive statistics presented.

#### Covariates

All covariates used in the analyses were derived from the parental portion of the survey. Age group was defined as 9–13 years and 14–17 years. Response options for sex at birth included male and female. Region of residence included West (British Columbia, Alberta, Saskatchewan and Manitoba), Ontario, Quebec and East (Newfoundland and Labrador, Nova Scotia, Prince Edward Island, and New Brunswick). Participant race/ethnicity was derived from the parental question about their child’s race/ethnicity, which was a ‘mark all that apply’ question. Responses included Black, East/Southeast Asian, Indigenous, Latino, Middle Eastern, South Asian, White, ‘Other’ (response option ‘another race’) and ‘Mixed’, when parents chose two or more race options. Annual household income response options included less than $20 000, $20 000 to less than $50 000, $50 000 to less than $80 000, $80 000 to less than $100 000, $100 000 or more and Don’t know/Prefer not to answer.

Finally, youth participants were asked what their gender was, with response options ‘Boy’, ‘Girl’ and ‘I identify my gender as: ____’. Given that only seventeen participants (1·1 %) identified their gender as something other than boy or girl, all results are presented in relation to sex at birth (male and female).

### Ethics

This study received ethics approval from Health Canada (REB 2021-017H) as well as University of Waterloo (#43578).

### Analysis

#### Descriptive statistics

Descriptive statistics (e.g. mean and standard deviations or proportions) were used to describe the distribution of sociodemographic characteristics and outcomes. Bar charts were used to visually display data related to appealing marketing features by age group.

Unadjusted and adjusted binary logistic regression models were fitted to examine associations between frequency of advertisement exposure and coviariates with *high-frequency consumption* and *restaurant spending*. Multi-collinearity and influential outliers were checked, and the Homer–Lemeshow test was used to evaluate model fit. Assumptions of linear regressions were tested, and unadjusted and adjusted linear regression models were fitted to examine associations between *frequency of advertisement exposure* and covariates with *money spent at restaurants*. For adjusted models, complete case analysis was used. Finally, we tested for effect modification by age group in both main regression models to evaluate whether the strength of relationships between variables were different for children *v*. adolescents.

All statistical analyses were conducted in SPSS version 28·0, and statistically significant findings were considered those with a *P* value < 0·05.

## Results

Table 1 shows outcomes of interest by sociodemographic characteristics of participants. A total of 42·8 % of participants were *high- frequency consumers*, 60·7 % of participants reported *restaurant spending (yes/no)* and the mean *money spent at restaurants* was $20·70 in the last 7 d. *High-frequency consumption* ranged from a low of 32·5 % of participants in Quebec to a high of 48·9 % in the West. In terms of *frequency of advertisement exposure*, 29·4 % of participants self-reported restaurant advertisement exposure at least once per day, 43·1 % reported seeing or hearing restaurant advertisements once per week to a few times per week, 18·5 % reported exposure to restaurant ads less than once per week and 9·0 % said they ‘Don’t know’ how often they saw or heard advertisements for restaurants in a typical week.

**Table 1:** Sample characteristics and outcomes of interest among children and youth aged 9–17 years living in the ten Canadian provinces in 2022 (*n* 1500)

	*n*	%	High-frequency restaurant consumption (> twice/week) (%)	Any money spent at restaurants in the past 7 d (%)	Dollar amount spent at restaurants among participants reporting > $0 in the past 7 d	
					Mean	sd
Total	1500	100 %	42·8	60·7	20·70	17·79
Sex						
Male	770	51·3	42·5	59·7	21·10	18·23
Female	730	48·7	43·2	61·7	20·28	17·33
Region^*^						
West	489	32·6	48·9	63·8	21·75	18·26
Ontario	592	39·5	43·6	65·5	21·24	19·24
Quebec	326	21·7	32·5	45·8	18·15	13·41
East	93	6·2	41·9	66·7	20·76	18·57
Age group						
9–12 years	659	43·9	37·8	50·8	20·48	17·75
13–17 years	841	56·1	46·7	68·5	20·88	17·84
Annual household income						
Less than $20 000	55	3·7	32·7	56·4	18·69	15·17
$20 000 to less than $50 000	219	14·6	39·7	60·1	18·63	17·17
$50 000 to less than $80 000	270	18·0	47·0	67·0	20·70	18·06
$80 000 to less than $100 000	245	16·3	47·8	60·6	21·39	18·38
$100 000 or more	600	40·0	42·7	60·5	20·96	18·04
Don’t know/prefer not to answer	111	7·4				
Race/ethnicity (missing *n* 20)						
Black	43	2·9	79·1	82·1	24·35	21·50
East or Southeast Asian	133	8·9	54·9	62·0	23·45	18·17
Indigenous	38	2·5	65·8	70·3	24·23	15·40
Latino	26	1·0	69·2	73·1	27·74	18·62
Middle Eastern	22	1·5	54·5	61·9	27·40	21·32
South Asian	84	5·5	42·2	74·4	19·83	20·38
White	986	65·7	37·3	58·4	19·79	17·20
Other	33	2·2	45·5	62·5	17·90	16·04
Mixed^†^	116	7·7	49·1	57·9	21·76	18·22

* Region: West included the provinces of British Columbia, Alberta, Saskatchewan and Manitoba, while East included the provinces of Nova Scotia, Prince Edward Island, Newfoundland and Labrador, and New Brunswick.

† Mixed race/ethnicity refers to participants whose parents checked at least two race/ethnicity options in the survey question.

Aligning with research objective 1, Table 2 shows logistic regression analysis of variables related to the odds of (1) *high-frequency consumption* and (2) *restaurant spending (yes/no).* Compared to participants who reported infrequent exposure to restaurant advertisements, participants who reported higher frequency of exposure had higher odds of being a *high-frequency consumer* in adjusted models (AOR = 2·20 95 % CI 1·60, 3·04 for ‘once a week to a few times per week’ and AOR = 2·37 95 % CI 1·80, 3·43 for ‘at least once a day’). Similarly, compared to participants who reported infrequent restaurant advertisement exposure, those who reported higher frequency of exposure had higher odds of *restaurant spending (yes/no)* in adjusted models (AOR = 1·62, 95 % CI 1·19, 2·20 for ‘once a week to a few times per week’, AOR = 1·77, 95 % CI 1·27, 2·47 for ‘at least once a day’).

**Table 2:** Associations between frequency of reported exposure to restaurant advertisements and frequently (> twice per week) consuming food or beverages from restaurants in the last 7 d, or of spending any money at restaurants among children and youth aged 9–17 years living in Canadian provinces in 2022 (*n* 1500)

Predictor	High-frequency restaurant consumption (> twice/week)				Spending any money at restaurants in the past 7 d			
	OR unadjusted	95 % CI	OR adjusted (*n* 1348)	95 % CI	OR unadjusted	95 % CI	OR adjusted (*n* 1327)	95 % CI
Frequency of reported exposure to restaurant ads								
Less than once per week (ref)	1·0		1·0		1·0		1·0	
Once a week to a few times/week	**2·13**	1·57, 2·89*	**2·20**	**1·60, 3·04**	**1·61**	**1·21, 2·14**	**1·62**	**1·19, 2·20**
At least once a day	**2·48**	**1·80, 3·43**	**2·37**	**1·69, 3·34**	**1·95**	**1·43, 2·66**	**1·77**	**1·27, 2·47**
Sex								
Male (ref)	1·0		1·0		1·0		1·0	
Female	1·03	0·84, 1·26	0·96	0·77, 1·20	1·09	0·88, 1·34	1·07	0·84· 1·35
Region								
Ontario (ref)	1·0		1·0		1·0		1·0	
West (BC, AB, SK, MB)	1·24	0·97, 1·57	1·26	0·96, 1·65	0·92	0·72, 1·20	0·99	1·74, 1·31
Quebec	**0·62**	**0·47, 0·83**	0·79	0·57, 1·08	**0·45**	**0·34, 0·59**	**0·46**	**0·34, 0·63**
East (NS, PE, NL, NB)	0·94	0·61, 1·46	1·11	0·68, 1·81	1·05	0·66, 1·67	0·98	0·59, 1·64
Age group								
Age 9–12 years (ref)	1·0		1·0		1·0		1·0	
Age 13–17 years	**1·44**	**1·17, 1·78**	**1·45**	**1·15, 1·82**	2·11	1·70, 2·61	**2·16**	**1·71, 2·73**
Income								
Less than $20 000	0·65	0·36, 1·17	1·04	0·64, 2·01	0·84	0·48, 1·48	1·03	0·53, 1·99
$20 000 to less than $50 000	0·89	0·65, 1·21	0·86	0·61, 1·22	0·98	0·72, 1·35	0·97	0·68, 1·38
$50 000 to less than $80 000	1·19	0·89, 1·59	1·17	0·85, 1·61	1·33	0·98, 1·80	1·29	0·92, 1·81
$80 000 to less than $100 000	1·23	0·91, 1·66	1·23	0·88, 1·71	1·00	0·74, 1·37	0·96	0·68, 1·36
$100 000 or more (ref)	1·0		1·0		1·0		1·0	
Don’t know/prefer not to answer	0·67	0·44, 1·03	**0·48**	**0·28, 0·82**	**0·65**	**0·43, 0·99**	0·67	0·42, 1·09
Race/ethnicity								
White	1·0		1·0		1·0		1·0	
Black	**6·34**	**3·01, 13·38**	**5·84**	**2·68, 12·73**	3·25	1·42, 7·44	**2·52**	**1·07, 5·93**
East or Southeast Asian	**2·04**	**1·42, 2·94**	**1·73**	**1·16, 2·60**	1·16	0·80, 1·69	0·95	0·62, 1·46
Indigenous	**3·23**	**1·63, 6·39**	**2·77**	**1·33, 5·76**	1·68	0·82, 3·44	1·41	0·64, 3·14
Latino	**3·78**	**1·63, 8·78**	**3·49**	**1·47, 8·32**	1·93	1·80, 4·63	1·97	0·76, 5·13
Middle Eastern	2·02	0·86, 4·71	2·00	0·81, 4·92	1·15	0·48, 2·81	0·88	0·35, 2·23
South Asian	1·23	0·78, 1·93	1·23	0·75, 2·04	**2·07**	**1·24, 3·45**	1·76	0·99, 3·13
Other	1·40	0·70, 2·81	1·25	0·57, 2·73	1·19	0·57, 2·45	1·20	0·52, 2·78
Mixed	**1·62**	**1·10, 2·39**	**1·67**	**1·09, 2·54**	0·98	0·66, 1·45	0·85	0·55, 1·32

* Bolded values mean statistically significant at *P* < 0·05.

Table 2 also describes differences in *high-frequency consumption* and *restaurant spending* outcomes by sex, region, age group, household income and race/ethnicity. Of note, there were no sex differences in either outcome. Youth had higher odds of being a *high-frequency consumer* and of *restaurant spending (yes/no)* relative to children in adjusted models (AOR = 1·45, 95 % CI 1·15, 1·82; AOR 2·16, 95 % CI 1·71, 2·73, respectively). Household income was not significantly related to either outcome, with the exception that participants whose household income was reported as ‘Don’t know/prefer not to answer’ had lower odds of being a *high-frequency consumer* relative to participants whose parents reported incomes of $100 000 per year or more (AOR = 0·48, 95 % CI 0·28, 0·82). There were several significant differences by race/ethnicity. For example, in adjusted models, relative to participants whose parents reported they were White, participants whose parents reported they were Black had higher odds of being a *high-frequency consumer* (AOR = 5·84, 95 % CI 2·68, 12·73) and of *restaurant spending* (AOR = 2·52, 95 % CI 1·07, 5·93). Similarly, in adjusted models, participants whose parents reported they were East or Southeast Asian, Indigenous, Latino and Mixed race all had higher odds of being *high-frequency consumers* relative to participants whose parents reported they were White.

Also aligned with research objective 1, Table 3 shows linear regression analysis of variables related to *money spent at restaurants*. In many ways, findings in Table 3 mirror those in Table 2. Participants who reported exposure to restaurant ads at least once per day spent significantly more money at restaurants in adjusted models relative to those who reported seeing or hearing restaurant ads less than once per week ($3·66, 95 % CI 0·89, 6·43). Children and youth whose parents reported they were Latino spent $8·05 (95 % CI $0·87, $15·23) more in restaurants than participants whose parents reported they were White. In adjusted models, there were no significant differences in *restaurant spending* by sex, age group, household income or region.

**Table 3:** Associations between frequency of reported restaurant advertisement exposure and the amount of money spent in the previous 7 d among children and teens aged 9–17 years living in Canadian provinces in 2022 who reported spending money at restaurants in the past 7 d (*n* 580)

Predictor	Mean spent at restaurants among participants reporting > $0 in the past 7 d^*^	sd	Unadjusted coefficient	95 % CI	Adjusted coefficient	95 % CI
Total	20·70	17·79				
Frequency of reported exposure to restaurant ads						
Less than once per week (ref)	19·27	16·94	–		–	
Once a week to a few times per week	19·53	17·94	0·26	−2·28, 2·79	0·06	−2·53, 2·64
At least once a day	23·38	18·69	**4·10**	**1·40, 6·81** ^†^	**3·66**	**0·89, 6·43**
Sex						
Male	21·10	18·23	–		–	
Female	20·28	17·33	−0·82	−2·62, 0·99	−0·95	−2·88, 0·98
Region						
Ontario (ref)	21·24	19·24	–		–	
West (BC, AB, SK, MB)	21·75	18·26	0·51	−1·62, 2·63	0·50	−1·81, 2·82
Quebec	18·15	13·41	**−3·09**	**–5·49, –0·69**	−2·47	−5·13, 0·19
East (NS, PE, NL, NB)	20·76	18·57	−0·49	−4·37, 3·40	0·50	−1·81, 2·82
Age group						
Age 9–12 years	20·48	17·75	–		–	
Age 13–17 years	20·88	17·84	0·41	−1·41, 2·22	0·09	−1·86, 2·05
Income						
Less than $20 000	18·69	15·17	−2·27	−7·18, 2·65	−1·78	−7·31, 3·75
$20 000 to less than $50 000	18·63	17·17	−2·33	−5·08, 0·43	−2·32	−5·29, 0·66
$50 000 to less than $80 000	20·70	18·06	−0·26	−2·81, 2·30	−0·01	−2·75, 2·74
$80 000 to less than $100 000	21·39	18·38	0·43	−2·22, 3·07	1·17	−1·68, 4·01
$100 000 or more (ref)	20·96	18·04	–		–	
Don’t know/prefer not to answer			1·94	−1·67, 5·54	1·77	−2·32, 5·86
Race/ethnicity (missing *n* 20)						
White	19·79	17·20	–		–	
Black	24·35	21·50	4·56	−0·86, 9·97	3·72	−2·04, 9·48
East or Southeast Asian	23·45	18·17	**3·66**	**0·45, 6·87**	2·83	−0·71, 6·37
Indigenous	24·23	15·40	4·44	−1·31, 10·18	3·53	−2·56, 9·62
Latino	27·74	18·62	**7·95**	**1·05, 14·86**	**8·05**	**0·87, 15·23**
Middle Eastern	27·40	21·32	**7·61**	**0·11, 15·10**	5·79	−2·04, 13·61
South Asian	19·83	20·38	0·04	−3·93, 4·016	−0·54	−4·88, 3·81
Other	17·90	16·04	−1·89	−8·04, 4·27	−4·17	−10·95, 2·61
Mixed	21·76	18·22	1·97	−1·45, 5·38	1·31	−2·38, 5·00

* Mean based on participants who reported spending up to $100 in the previous 7 d (*n* 21 participants excluded for reporting > $100).

† Bolded values mean statistically significant at *P* < 0·05.

**Figure 1:**
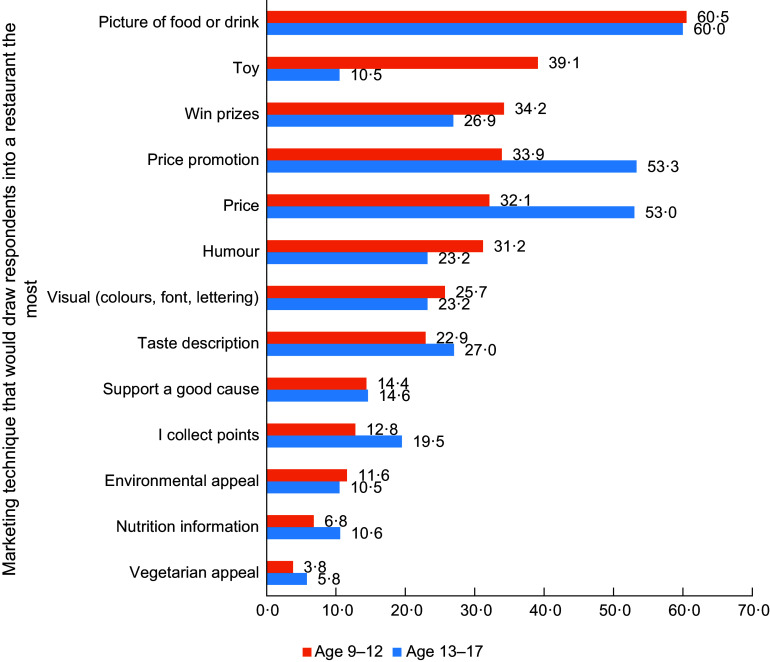
Percent of respondents reporting that a given marketing technique would ‘draw them into a restaurant the most’ by age group (*n* 1500 children and youth aged 9–17 years living in the ten Canadian provinces, 2022).

Aligned with research objective 2, Figure 1 presents findings related to *appealing marketing techniques of restaurant ads* by age group. Pictures of foods or drinks was the most frequently reported marketing technique for both age groups (60·5 % of children and 60·0 % of youth). The largest discrepancies by age group were those related to price, with 53·3 % and 53·0 % of youth participants indicating that price promotions and the price on an advertisement would draw them into a restaurant the most, compared to only 33·9 % and 32·1 % of children, respectively. There were also large discrepancies in the appeal of toys (39·1 % of children *v*. 10·5 % of youth), humour (31·2 % of children *v*. 23·2 % of youth) and winning prizes (34·2 % of children *v*. 26·9 % of 13–17 youth). The three most infrequently selected marketing techniques were appeals regarding vegetarian options, that items were good for the environment and availability of nutrition information.

All tests for effect modification by age group were not statistically significant.

## Discussion

This study examined whether and to what extent reported frequency of restaurant advertisement exposure from all sources was associated with (i) frequency of restaurant food and beverage consumption and (ii) money spent at restaurants in the past week in a sample of children and youth (aged 9–17 years), representative of the Canadian population by age, sex and region. This study also described the restaurant marketing techniques children and youth reported to be appealing. Three main findings emerged. First, overall, 43 % of participants consumed restaurant foods and drinks more than twice per week, 61 % spent at least some money at a restaurant in the last 7 d, and of those who spent money, the mean expenditure in the last 7 d was $20·70. Second, frequency of self-reported restaurant advertisement exposure was significantly associated with all outcomes, and several significant differences in outcomes emerged by region, age and race/ethnicity. Third, with regard to age differences, pictures of foods and drinks were the most frequently noted appealing marketing technique among both age groups; however, there were large discrepancies in perceptions about the following appealing advertisement features by age: price and price promotions, toys, humour, and winning prizes. Each of these findings is described in greater detail below.

First, findings from this study align with Canadian^(35)^ and international^(8,9)^ research findings on the high frequency of restaurant patronage among youth. Our participants were representative of the Canadian population by age, sex and region of residence. The fact that 43 % of participants (47 % of youth participants) reported eating or drinking restaurant foods and beverages more than twice a week is concerning, given the poor nutritional quality of restaurant foods, and children’s meals in particular^(11,36)^. We are unaware of other research identifying the amount of money spent at restaurants by children and teens. Over half of participants (61 %) reported spending at least some money in the last 7 d at a restaurant and of those who did, the mean expenditure was just over $20. Given the relatively low cost per calorie of fast food in particular^(37)^, $20 could potentially buy two or three meals over the course of a week. Of note, the survey items to assess spending at restaurants asked youth to identify whether and how much money *they themselves* spent, so the estimates provided in this paper are likely an underestimate, given that parents may have also spent family money on the children for restaurant food, which the survey did not capture.

Second, about half (51 %) of respondents reported being exposed to restaurant advertisements a few times per week or less. Given that restaurant advertising comprises 22 % of digital exposure (which has been estimated at more than 4000 advertisements per year for children and more than 8000 advertisements per year for teenagers)^(26)^, and that children in Canada are exposed to more than 1100 restaurant advertisements per year on television alone^(38)^, reported exposure among our participants was likely an underestimate. Despite this, self-reported frequency of restaurant advertisement exposure was still associated with high-frequency restaurant consumption, restaurant spending and with the overall amount of money youth spent at restaurants. This finding was not surprising given the well-established causal relationship between exposure to marketing and children’s food preferences/choices and food intake, indicating the negative health and nutritional impacts of unhealthy food marketing on children and youth^(17–22)^.

Third, differences in outcomes were observed by region and race/ethnicity, but no sex differences were noted. Notably, in Quebec, youth had about half the odds of spending any money at a restaurant in the past 7 d compared to those living in Ontario in the adjusted model. Interestingly, Quebec is the only province that restricts all commercial advertising directed to children under 13 years, including food advertising^(39)^, which may have explained the differences between Quebec and Ontario where advertising is self-regulated by the food and beverage industry. Research has previously found that Quebec’s advertising restrictions were associated with a decrease in the likelihood of purchasing fast food among Francophone households with children^(39)^.

In terms of race/ethnicity, participants whose parents identified them as Black, East or Southeast Asian, Indigenous or Latino had significantly higher odds of being high-frequency restaurant consumers compared to participants whose parents identified them as White, independent of differences in restaurant marketing exposure. This was the first study to suggest racial/ethnic differences in the frequency of restaurant food consumption in Canada, which contributes to a broader literature on racial/ethnic differences in outcomes related to diet. While racial/ethnic differences in dietary intake are frequently observed in the USA, very little research to date has explored racial/ethnic differences in dietary patterns among Canadian residents (especially children and youth)^(40)^. One recent study among Canadian adults found that although racial identity was not independently associated with diet quality, racial identity and perceived income adequacy interacted to jointly shape diet quality^(41)^. Other recent Canadian research has also found racial/ethnic differences in reported youth exposure to online unhealthy food marketing^(42)^; however, this was the first study to identify racial/ethnic differences in the frequency of restaurant food consumption. In 2020, Fischer and colleagues^(43)^ suggested several public health policy options to address both the systematically higher advertising of unhealthy dietary choices to Black and Hispanic youth in the USA and the systematically higher rates of diet-related chronic disease among these youth. Policy options included increasing the availability of affordable, nutritious foods in underserved neighbourhoods, regulating the quality of food advertised to minority youth and using multi-level educational dietary interventions to address these disparities. In Canada, however, racial/ethnic disparities in food and beverage marketing to children and in dietary or diet-related health outcomes are less clear. Therefore, future research should monitor racial/ethnic differences in both marketing and diet-related health outcomes over time.

Finally, this study identified particularly appealing restaurant marketing techniques among Canadian children and youth and found that while pictures of foods and drinks were the most frequently reported appealing marketing technique for 60 % of participants, there were large discrepancies by age group for price/price promotions, with 53 % of those aged 13–17 years reporting these techniques appealed to them whereas under a third of those aged 9–12 years reported similarly. On the other hand, toys, humour and winning prizes were much more popular among the younger age group. These findings align with recent work exploring the persuasive power of food and beverage marketing among teens^(44)^. Specifically, research with teens found ‘visual style’ (aligning with our findings on pictures of foods and drinks), ‘special offer’ (aligning with our findings on price promotions, toys and winning prizes) and ‘humour’ were in the top five indicators of teen-appealing ads^(44)^. For policies aiming to curtail unhealthy food marketing to children, regulating ‘visual style’ may prove to be a difficult task, even though it was the most frequently mentioned appealing advertisement feature in the current study and has been found to be one of the most appealing marketing techniques for teens^(44)^. Notably, while our survey question to assess students’ perceptions on appealing advertisement features were not designed to be open-ended, the pre-defined response options emerged directly out of a qualitative study with twenty-seven Canadian youth from across the country who were in the same age range.

Recently, Health Canada proposed regulations to limit the advertising of foods and beverages high in sugar, fat and salt that are ‘primarily directed’ to children under 13 years on television and digital media^(45)^. While a compulsory regulatory approach is a step in the right direction and in line with WHO guidance^(46)^, our findings raise questions about the likely effectiveness of advertising restrictions that focus exclusively on advertising that is ‘primarily directed’ at children. As our study found, ‘pictures of food and drinks’ were identified as the most appealing content in restaurant marketing by children aged 9–12 years. However, such content cannot be construed as uniquely appealing or directed to children of this age. The same could be said about characterising price promotions as ‘primarily directed’ to adolescents particularly as these are likely to be broadly appealing and directed to most consumers. Overall, these findings highlight probable gaps in regulatory/policy approaches focused on limiting advertisements ‘primarily directed’ to young people and lend support for broader restrictions that would limit children’s overall exposure to unhealthy food marketing, as recommended by the WHO’s most recent guidelines^(46)^.

Strengths of this study included the large sample size that was representative of the Canadian population living in the ten provinces (99·7 % of Canada’s population)^(34)^ by sex, region of residence and age. As noted, it was among the first studies to specifically explore restaurant marketing and consumption among a national sample of youth, which fills an important gap in the literature given the substantial contribution of restaurant foods to children’s and teen’s diets globally. Limitations include the cross-sectional nature of the study, which precludes causal attributions. It is also possible that participants who spent more money on or consumed more restaurant foods were also more aware of restaurant advertising. In addition, the non-probabilistic sample limits the generalisability of these findings, and the lack of inclusion of participants from Canada’s three Northern Territories limits the representativeness of our sample relative to the Canadian population as a whole. Moreover, data were self-reported rather than measured, and it is possible that data quality from younger participants was poorer given their stage of cognitive development relative to older participants. In addition, participants were not able to generate their own responses to closed-ended questions, meaning that our survey may not have captured all possible advertisement features that youth find appealing. Although survey questions were not tested for validity or reliability, they were informed by a prior qualitative study with twenty-seven Canadian youth from multiple provinces.

### Conclusion

In conclusion, a large proportion of Canadian children and youth consumed restaurant offerings more than twice a week, which may have detrimental impacts on their overall diet and health. Moreover, their reported frequency of exposure to restaurant advertising was significantly positively associated with restaurant consumption frequency, whether or not they spent any money at a restaurant in the last 7 d, and the amount of money they spent at restaurants (among youth who reported spending money).
